# Recreational Cannabis Legalization in the US and Maternal Use during the Preconception, Prenatal, and Postpartum Periods

**DOI:** 10.3390/ijerph17030909

**Published:** 2020-02-01

**Authors:** Kara R. Skelton, Amelie A. Hecht, Sara E. Benjamin-Neelon

**Affiliations:** 1 Department of Health, Behavior, and Society, Johns Hopkins Bloomberg School of Public Health, 624 North Broadway Baltimore, MD 21205, USA; Sara.neelon@jhu.edu; 2 Department of Health Policy and Management, Johns Hopkins Bloomberg School of Public Health, 624 North Broadway Baltimore, MD 21205, USA; ahecht3@jhu.edu

**Keywords:** marijuana, perinatal health, substance use, PRAMS

## Abstract

In the United States (US), recreational cannabis use is on the rise. Since 2011, 11 states and the District of Columbia have legalized cannabis for adult recreational use. As additional states consider legalizing, there is an urgent need to assess associations between recreational cannabis legalization and maternal use in the preconception, prenatal, and postpartum periods—all critical windows for maternal and child health. Using cross-sectional data from the 2016 Pregnancy Risk Assessment Monitoring System, we assessed associations between state cannabis legalization and self-reported maternal cannabis use. Using logistic regression, we estimated the adjusted prevalence ratio (PR) of cannabis use during the preconception, prenatal, and postpartum period for women delivering a live-born infant in three states that had legalized recreational cannabis (Alaska, Colorado, and Washington) and three states that had not legalized (Maine, Michigan, and New Hampshire) by 2016. Our final sample size was 7258 women. We utilized 95% confidence intervals (CI) and a significance level of *alpha* = 0.05. After adjustment for potential confounders, women who resided in states with legalized recreational cannabis were significantly more likely to use cannabis during the preconception (PR 1.52; 95%CI ranging from 1.28–1.80; *p* < 0.001), prenatal (PR 2.21; 95% CI ranging from 1.67–2.94; *p* < 0.001), and postpartum (PR 1.73; 95%CI ranging from 1.30–2.30; *p* < 0.001) periods, compared to women who resided in states without legalized recreational cannabis. Although evidence about the effect of marijuana use during these periods is nascent, these findings show potential for increased incidence of child exposure to cannabis. Longitudinal research is needed to assess immediate and sustained impacts of maternal use before and after state legalization of recreational cannabis.

## 1. Introduction

Use of recreational cannabis in the United States (US) by women of reproductive age and pregnant women is increasing [1,2,3]. Recent prevalence estimates of past-month recreational cannabis use among US pregnant and nonpregnant women in 2017 were 4.03% and 8.73%, respectively [3]. Although cannabis is federally classified as an illegal Schedule I drug under the Controlled Substances Act [4], 11 states and the District of Columbia have legalized cannabis for adult recreational use. Numerous other states, including New York, Hawaii, and Minnesota considering bills to legalize recreational cannabis in 2020 [5]. Additionally, 23 states and the District of Columbia have legalized medicinal cannabis. There may be benefits to legalizing recreational cannabis [6], including positive criminal justice outcomes [7] and economic investment in local communities [8]. However, there are also public health concerns of legalizing the drug, including impaired driving while under the influence of cannabis [9] and increased incidence of accidental child exposure to cannabis [10,11]. Thus, it is imperative that the potential impact of cannabis legalization on vulnerable groups like infants and young children be considered when deliberating recreational cannabis legislation [11,12,13], as has been done in other countries that have legalized recreational cannabis at a national level [14,15]. 

Increases in maternal cannabis use, during the preconception, prenatal, and postpartum periods, in combination with increasing THC potency and product concentrations, elicit concern about potential adverse health consequences for both women and children [10,11,12,16,17,18]. In the US, prevalence estimates of past-month prenatal cannabis use have escalated from 3.4% in 2002 to 7.0% in 2016 [3]. Increase use of cannabis during pregnancy and postpartum may have implications for maternal and child health, but these outcomes are not yet well understood. Studies examining the relationship between perinatal cannabis use and adverse child health outcomes show mixed findings [1,19,20,21,22,23,24,25,26,27,28]. Emerging research indicates the female reproductive system is influenced by the endocannabinoid system, meaning chronic exposure to cannabis could lead to disruption of menstrual cycles, depression of ovarian follicular maturation, and reductions in key hormones needed for ovulation [29]. The pharmacokinetics of other substances in cannabis, such as cannabidiol (CBD), during the preconception, prenatal, and postpartum periods have been scarcely studied [30]. Additional research indicates that CBD influences estrogen signaling, crosses through the placenta and is transferred into breast milk [31,32]. One systematic review reported associations between prenatal cannabis use and negative birth outcomes, such as low birth weight and neonatal intensive care unit admissions [33], whereas another review found no association [22]. The American College of Obstetrics and Gynecology reports fetal growth restrictions, preterm birth, neonatal intensive care unit admission, and negative neurodevelopmental outcomes as adverse perinatal health outcomes of prenatal cannabis use [13]. A recent review of cannabis literature found evidence that THC inhibits the secretion of prolactin [34]: a necessary hormone in breast milk production. Most prior studies have methodological flaws, such as uncontrolled confounding, inability to determine independent effect of cannabis use, and lack of standardization of cannabis outcome measures [12,35]. Older studies also lack contemporary relevance due to the changing legality of the drug, increases in 11-hydroxy-∆-9-tetrahydrocannabinol (THC) potency over the past several decades [36], and new cannabis products and modes of administration. Federal classification of cannabis as a Schedule I substance, as well as ethical challenges in conducting randomized controlled trials with pregnant and breastfeeding women, limit the availability of rigorous experimental studies. Therefore, much of what we currently know regarding perinatal cannabis use is from observational studies. Combined, these limitations leave the scientific field cloudy for meaningful interpretation for both policy and clinical practice. 

Upward trends of maternal cannabis use increases appear to coordinate with increases in child exposure to cannabis [16]. THC crosses the human placenta, introducing the drug to the fetus in utero [13]. Children may be exposed to cannabis through additional mechanisms beyond in utero exposure, including breast milk consumption [32], second-hand smoke exposure [16] and accidental ingestion of cannabis products [11,17]. THC is a highly lipophilic compound that is readily transferred to breast milk and takes a substantial amount of time to be cleared, the estimated half-life of THC in breast milk to be roughly 27 hours [32]. Over the past decade, there has been an increase in pediatric exposure to cannabis that appears to be associated with the additional availability of cannabis-containing products and increases in cannabis users in the US [11,37]. Data from the US National Poison Data System for 2000–2013 showed a total of 1969 children <6 who were exposed to cannabis in the US, with 92.2% of these child cannabis exposures unintentional. Ingestion was the most common form exposure (75.0%) and second-hand inhalation was second, accounting for approximately 14.5% of all child cannabis exposures [16]. In Colorado, there was a 150% increase in regional poison control center cases and pediatric hospitalizations after recreational cannabis was legalized [37]. Polysubstance use is common among prenatal cannabis users and includes co-use of tobacco and alcohol [21,38,39], meaning increases in perinatal cannabis use may coincide with child exposure to multiple teratogens throughout critical developmental periods. Thus, perinatal cannabis use may have greater child health implications than what has been previously reported [12,36].

Although previous studies show that cannabis use is increasing over recent years for women of reproductive age [1], as well as during pregnancy [2,40], most prior studies have not assessed the relationship between this upward trend and recreational cannabis legalization. Studies that have examined this association have looked only at maternal cannabis use within a single state and time period [41]. It is important to examine how cannabis use varies for women who reside in states with and without legalized recreational cannabis. Therefore, this study aims to assess the relationship between state recreational cannabis legalization and maternal cannabis use across the preconception, prenatal, and postpartum periods using cross-sectional data comparing states where recreational cannabis was and was not legal. We aimed to test the hypothesis that women who resided in states with legalized cannabis would report substantially higher rates of cannabis use during the preconception, prenatal, and postpartum periods compared to women who resided in states that have yet to legalize recreational cannabis. 

## 2. Materials and Methods

We used cross-sectional data from the 2016 Pregnancy Risk Assessment Monitoring System (PRAMS), a surveillance system of the Centers for Disease Control and Prevention (CDC) that provides data on numerous maternal and child health indicators not available from other sources [42]. PRAMS collects data about maternal behavior and experiences before, during, and after pregnancy, covering approximately 83% of all live births in the US [42]. Participating PRAMS states develop questionnaires for administration on an annual basis, surveying women between 3 and 9 months postpartum about their experiences during the preconception, prenatal, and postpartum periods. States participating in PRAMS may optionally include questions about cannabis use. Upon request, the CDC provides PRAMS data to researchers for states that have met the 55% annual response rate threshold. Additional information about PRAMS methodology has been reported elsewhere [42]. 

We used data from the six states in 2016 that collected information about cannabis use: Alaska, Colorado, Maine, Michigan, New Hampshire, and Washington. In these six states, a total of 7258 women provided information on maternal cannabis use. We did not restrict the sample based on any prenatal or birth outcomes (e.g., gestational diabetes, singleton only). There were 3182 and 4076 women in states where recreational cannabis was illegal and in states where recreational cannabis was legal, respectively. All six states had legalized medicinal marijuana at the time of data collection. The CDC Institutional Review Board approved this secondary data analysis; the Johns Hopkins Bloomberg School of Public Health Institutional Review Board determined that this research was exempt.

### 2.1. Measures

#### 2.1.1. Cannabis Use

Cannabis use was defined as an affirmative response to the question, “Did you use marijuana or hash in any form?” Responses to this question allowed the creation of three outcomes variables: (1) cannabis use in the 12 months prior to the most recent pregnancy (preconception), (2) cannabis use during the most recent pregnancy (prenatal), or (3) cannabis use since the most recent baby was born (postpartum).

#### 2.1.2. Recreational Cannabis Legalization

We created an indicator variable representing if recreational cannabis for adult use (21+ years) was legal (Alaska, Colorado, Washington) or illegal (Maine, Michigan, New Hampshire) in 2016. Alaska legalized recreational cannabis in 2014; both Colorado and Washington legalized recreational cannabis in 2012. Recreational cannabis was illegal in Maine, New Hampshire, and Michigan at the time of data collection. Although Maine voted to legalize recreational cannabis in November 2016; this law did not go into effect until January 2017 date. Michigan also legalized recreational cannabis in 2018. Recreational cannabis currently remains illegal in New Hampshire. 

#### 2.1.3. Medicinal Cannabis Legalization

Medical cannabis was legal in all states included in this analysis. However, there were varying restrictions regarding possession and cultivation limits, as well as regulation. Most states delineated specific conditions for which medical cannabis was an approved treatment. These included terminal and debilitating diseases and select other chronic conditions. At the time of data collection, Alaska, Colorado, and New Hampshire did not have state-regulated medical cannabis dispensaries. Michigan and New Hampshire did not allow dispensaries of any kind in 2016; Maine allowed only one medical cannabis dispensary per district (eight total) in 2016. However, in states where cannabis was legal, medical cannabis could also be purchased at retail dispensaries, if the state allowed retail dispensaries.

### 2.2. Analysis

We calculated prevalence of maternal cannabis use during the preconception, prenatal, and postpartum periods. We used multivariate logistic regression and postestimation commands with linearized standard errors to estimate adjusted prevalence ratios (PRs) of maternal cannabis use during the preconception, prenatal, and postpartum periods. We adjusted a priori for variables available in the PRAMS data that have been found to be associated with cannabis use based on contemporary studies [43,44,45]. These included maternal age (≤17, 18–24, 25–34, 35+ years), race (white, not white), ethnicity (Hispanic, not Hispanic), education (completed high school or less, completed more than high school), marital status (married, not married), household income (<$40,000, ≥$40,000), participation in the Special Supplemental Nutrition Program for Women, Infants and Children (yes, no), health insurance status (public, private/military, no insurance), whether women initiated prenatal care during the first trimester (yes, no), and cigarette smoking during the corresponding time period (yes, no). We further adjusted for breastfeeding for the postpartum analysis only (yes, no). We included state-specific survey weights for the complex sampling design, non-response, and non-coverage in all analyses. We present adjusted PR and 95% confidence intervals (CI), using a significance level of *alpha* = 0.05. We conducted all analysis using Stata 14.1 (StataCorp, College Station, TX, USA). 

## 3. Results

The final analytic cohort was 7258 women. Table 1 reports descriptive characteristics of the overall sample, as well as maternal and household characteristics by state recreational cannabis legality. 

### 3.1. Sample Characteristics

### 3.2. Maternal Cannabis Use

Overall weighted cannabis use estimates were 14.73% in the preconception, 5.65% in the prenatal, and 6.45% in the postpartum periods (Table 2). Women who resided in states with legalized recreational cannabis reported higher prevalence of cannabis use in each period, compared to women residing in states without legalized recreational cannabis (Table 2).

### 3.3. Maternal Cannabis Use

In adjusted models, women in states that had legalized recreational cannabis were more likely to use cannabis during each time period than women in states where recreational cannabis was illegal. On average, compared to women living in states where recreational cannabis was illegal, women residing in states where recreational cannabis was legal were 1.52 times more likely to use cannabis during the preconception period, 2.21 times more likely to use cannabis during pregnancy, and 1.73 times more likely to use cannabis in the postpartum period (Table 3).

## 4. Discussion

Using 2016 cross-sectional data from PRAMS, we were able to calculate recent perinatal cannabis use prevalence estimates as well as provide a meaningful picture of how maternal cannabis use compares across states with and without recreational cannabis legalization. Specifically, we found that prevalence and adjusted likelihood of maternal cannabis use during three critical developmental periods was significantly higher in states where recreational cannabis is legal compared to states where recreational cannabis was not legal. This is the first study is the first to explore the association between recreational cannabis legalization and maternal cannabis use across all three of these periods across multiple states.

Self-reported prenatal cannabis use rates in states with legalized recreational cannabis in this analysis were similar (6.76%) compared to an examination of 2014–2015 Colorado PRAMS data (5.7%) [44]. We found slightly higher rates of prenatal cannabis use in states that had not legalized compared to previous studies. Before recreational cannabis was legal in the US, researchers using 2007–2012 data from National Surveys of Drug Use and Health (NSDUH), a nationally-representative surveillance system [40] found that 3.9% of pregnant women reported past-month cannabis use. The higher rates of self-reported prenatal cannabis use reported in our study compared to others [1] may be due to increased perceived safety of the drug, as pregnant women in one study reported a belief that prenatal cannabis use was save to treat nausea [46]. Perhaps the inclusion of multiple states with and without legalized recreational cannabis in our study, or differences in mode of data collection (e.g., surveillance system, self-report vs. toxicology screen) is responsible for variations in prevalence estimates across studies. Or, as it takes time to see changes after policies are enacted, it could be that in relation to data collection, these prior studies had not allowed enough time to pass to detect changes in maternal cannabis use [47].

Compared to other studies that used objective measures (e.g., urine toxicology screen and umbilical cord sampling), the self-reported prenatal cannabis use estimates in states with legalized recreational cannabis were much lower. For example, using umbilical cord sampling, Metz et al. (2019) found prenatal cannabis use was 22.4%, 95%CI ranging from 15.2–31.1, over twice the estimates we calculated based on self-report [48]. Using both self-report and toxicology detection, Gnofam et al. (2019) found the prevalence of prenatal cannabis use trended higher after recreational cannabis legalization in Colorado and that no similar trend was observed for other substances, including tobacco and opioids [41]. A prior study found that approximately 18.1% of prenatal cannabis users meet criteria for cannabis abuse, dependence, or both [40], indicating that contemporary prenatal cannabis use may be reduced via interventions aimed at treating reasons for cannabis dependency. These findings, taken together with results of existing studies, indicate there maternal and child health outcomes may be impacted by changing recreational cannabis policies.

The results of this study indicate that additional longitudinal research is warranted to assess changes in maternal cannabis use patterns, both over time and in relation to recreational cannabis legalization. As this paper did not aim to examine impact of legalization, but rather associations, prospective research is needed. There are several ongoing cohort studies in the Netherlands [49] and Canada [50], which examine maternal cannabis use, as well as the impact of recreational cannabis legalization on adolescent behaviors [51]. Although these cohort studies will address significant gaps in the research, there is a need for similar prospective, population-based studies relating to maternal cannabis use and associated maternal and child health outcomes in the US. Feasibility of innovative and objective methodologies, such as umbilical cord sampling, as well as other noninvasive methods of perinatal cannabis screening should be examined. Additionally, future studies could consider examining maternal polysubstance use during these time periods to see whether women who use cannabis are also more likely to use other substances. These studies could also examine how state legalization of recreational cannabis impacts the use of other substances during the preconception, prenatal, and postpartum periods. 

Until such studies are undertaken, or until we see outcomes of existing population-based prospective cohort studies, policymakers and clinicians in states with legalized recreational cannabis should partner to provide non-punitive education about the potential impacts of cannabis use on maternal and child health, and referral to treatment, as needed, for women during these critical periods. An array of policy evaluation studies are also needed to determine what types of policies are most effective in protecting maternal and child health from cannabis exposure-related consequences. These include, but are not limited to, product availability, packaging (e.g., maximum amount of THC per packaged product and warning labels for pregnant and breastfeeding women), distribution (e.g., home delivery and on-site consumption only), home storage, and screening. In the meantime, states should work collaboratively to learn from one another—especially from states that legalized recreational cannabis earlier, in order to benefit from best practices and lessons learned. States could implement mass-reach health communication campaigns to educate residents on the “safe, legal, and responsible” use of cannabis, as was done in Colorado [52]. However, this public health campaign was implemented after recreational cannabis legalization took effect. Ultimately, it is imperative that programs, policies, and regulations designed to protect public health are implemented prior to, and not after, legalization.

We were limited by PRAMS data availability. Therefore, we were only able to include PRAMS states that asked cannabis questions and met the CDC response rate threshold for public data release (six states). The sampling and selection strategy for each PRAMS state varies annually, which limits our ability to make longitudinal associations with the same sample of women. Additionally, cannabis use data are self-reported and retrospective. As mentioned above, prior studies have shown that self-reported cannabis use during pregnancy was underreported when compared to toxicology [2]. Social desirability bias and potential fear of punitive action may have resulted in underreporting among the entire sample. However, this fear may have been particularly heightened among women who resided in states without recreational cannabis legalization, as well as those women under the age of 21, for whom recreational cannabis is not legal in any state. Additionally, although there was slight variation in the wording for the state-developed cannabis questions used by Colorado, all questions asked about preconception, prenatal, and postpartum “marijuana or hash” use. As a result, we were unable to examine different routes of cannabis administration and frequency of use based on wording of the PRAMS questions. Due to the cross-sectional nature of this study, causality cannot be determined. An additional limitation of this study is that medicinal marijuana was legal in all included states at the time of data collection, which could affect the findings of this analysis. However, the proportion of women using medical cannabis is very small nationally; recent data from the National Survey on Drug Abuse and Health (2013–2017) showed that past-month medicinal cannabis use among pregnant women was no more than 0.7% and past-month medicinal cannabis use among non-pregnant women was 1.1% [3]. Although we did control for many variables in this analysis, we were unable to control for effects of state differences of other variables that may affect cannabis use, due to variations in PRAMS sampling strategy at a state level. We also recognize that, importantly, states that legalized cannabis may be systematically different from states that have not legalized. However, Maine and Michigan have since legalized recreational cannabis and New Hampshire considered recreational cannabis legalization in 2019, indicating similar attitudes among policymakers and voters toward cannabis. 

## 5. Conclusions

This study examined associations between recreational cannabis legalization and maternal cannabis use across three critical maternal and child health periods. Despite limitations, results indicate that women in states with legalized recreational cannabis were more likely to use during the preconception, prenatal, and postpartum periods, compared to women in states where recreational cannabis was illegal. Given the current lack of sufficient evidence regarding effects of perinatal cannabis use on both maternal and child health outcomes, this study highlights the need for high-quality, longitudinal investigations that examine this relationship. However, in the absence of more robust evidence on the impact of changing cannabis policies on maternal and child health cannabis exposure, policy-makers, voters, and other stakeholders should be cautious about continued legalization of recreational cannabis.

## Figures and Tables

**Table 1:** Characteristics of sample by recreational cannabis legality in 2016 ^a^.

Characteristic	%Total (SE)	%Recreational Cannabis Illegal (Unweighted) (SE)	% Recreational Cannabis Legal (Unweighted) (SE)	*p*-Value
**n**	**7258**	**3182**	**4076**	
Married	67.92 (0.80)	61.85 (1.19)	72.87 (10.07)	<0.0001
Maternal age (years)				0.0001
≤17	1.11 (0.20)	1.33 (0.32)	0.94 (0.25)	
18–24	19.56 (0.69)	22.49 (1.08)	17.16 (0.88)	
25–34	61.10 (0.84)	60.07 (1.25)	61.95 (1.15)	
35+	18.22 (0.66)	16.11 (0.91)	19.95 (0.95)	
Household Income				0.0040
<$40,000	45.92 (0.85)	48.63 (1.25)	43.71 (1.15)	
≥$40,001	54.08 (0.85)	51.37 (1.25)	56.29 (1.15)	
Race				<0.0001
White	74.12 (0.57)	75.07 (0.81)	73.33 (0.80)	
Black	9.75 (0.28)	15.27 (0.20)	5.13 (0.49)	
Other Race	8.51 (0.47)	6.23 (0.68)	10.42 (0.64)	
Asian	6.07 (0.28)	3.13 (0.44)	8.53 (0.35)	
American Indian Alaskan Native	1.55 (0.11)	0.30 (0.15)	2.59 (0.16)	
Ethnicity				<0.0001
Hispanic	14.92 (0.50)	5.59 (0.68)	22.63 (0.71)	
Non-Hispanic	85.08 (0.50)	94.41 (0.68)	77.37 (0.71)	
Education				0.0188
Less than High School	10.23 (0.52)	9.60 (0.78)	10.74 (0.700)	
Completed High School	23.63 (0.77)	25.98 (1.16)	21.70 (1.03)	
Some College	28.58 (0.77)	28.74 (1.12)	28.44 (1.05)	
≥4-year College	37.56 (0.82)	35.68 (1.19)	39.11 (1.13)	
WIC Participation	35.11 (0.82)	36.82 (1.22)	33.69 (1.11)	0.06
Health Insurance Status ^b^				0.0001
Public	21.56 (0.71)	24.29 (1.07)	19.31 (0.94)	
Private/Military ^b^	68.88 (0.79)	67.93 (1.17)	69.67 (1.08)	
No Insurance	9.56 (0.50)	7.78 (0.69)	11.03 (0.72)	
Breastfeeding Status				<0.0001
Ever Breastfed	90.63 (0.51)	85.14 (0.94)	95.16 (0.51)	
Still Breastfeeding	65.47 (0.86)	59.01 (1.34)	70.27 (1.13)	
Cigarette Smoking				<0.0001
Preconception	18.59 (0.69)	21.98 (1.09)	15.83 (0.89)	
Prenatal	8.49 (0.50)	11.20 (0.85)	6.28 (0.59)	
Postpartum	12.32 (0.59)	15.66 (9.60)	9.60 (0.72)	
First Trimester Prenatal Care	88.51 (0.56)	87.66 (0.85)	89.21(0.74)	0.17

SE: standard error; WIC: Special Supplemental Nutrition Program for Women, Infants, and Children. ^a^ Includes Alaska, Colorado, Maine, Michigan, New Hampshire, and Washington (unweighted *n* = 7258). ^b^ Private insurance includes TRICARE or other military insurance, private health insurance, health insurance through parents, and health insurance through the health care exchange. Public insurance includes Medicaid, CHIP, Indian Health Service (Alaska only), or other government health insurance. “No insurance” included women who reported no insurance or Indian Health Service only (excluding Alaska).

**Table 2:** Unadjusted cannabis use prevalence by cannabis law status in 2016.

Cannabis Use Period	% Total (Unwieghted) (SE)	% Recreational Cannabis Illegal (Unweighted) (SE)	% Recreational Cannabis Legal (Unweighted) (SE)
**n**	**7258**	**3182**	**4076**
Preconception	14.73 (0.63)	13.46 (0.84)	15.78 (0.92)
Prenatal	5.65 (0.42)	4.29 (0.48)	6.76 (0.65)
Postpartum	6.45 (0.44)	5.84 (0.58)	6.95 (0.65)

**Table 3:** Unadjusted and adjusted ^a^ prevalence ratio and prevalence difference of cannabis use before, during, and after pregnancy by cannabis law status in 2016.

Cannabis Use Period	Unadjusted Prevalence Ratio (95%CI), SE	Unadjusted Prevalence Difference (95%CI), SE	Adjusted Prevalence Ratio (95%CI), SE	Adjusted Prevalence Difference (95%CI), SE	*p*-Value
Preconception	1.17 (0.99–1.39), 0.10	0.02 (−0.001–0.05), 0.01	1.52 (1.28–1.80), 0.13	0.06 (0.4–0.09), 0.01	<0.0001
Prenatal	1.58 (1.18–2.11), 0.23	0.03 (0.008–0.04), 0.008	2.21 (1.66–2.9), 0.32	0.05 (0.03–0.06), 0.009	<0.0001
Postpartum	1.19 (0.91–1.56), 0.16	0.01 (−0.006–0.03), 0.009	1.73 (1.30–2.30), 0.25	0.03 (0.02–0.05), 0.01	0.0003

CI: Confidence Interval, SE: standard error. ^a^ Logistic regression with postestimation commands, adjusted for race, ethnicity, marital status, education, household income, WIC participation, maternal age, health insurance status, cigarette smoking during corresponding time period, and received prenatal care during first trimester. We further adjusted for breastfeeding for the postpartum analysis only. We used survey weights to account for sampling strategy.
